# Reimagining André Rey's method for recording the copy process of the Rey Complex Figure Test: A commentary

**DOI:** 10.1111/jnp.12414

**Published:** 2025-01-24

**Authors:** Maneesh V. Kuruvilla, Angela Blazely

**Affiliations:** ^1^ Wicking Dementia Research and Education Centre University of Tasmania Hobart Tasmania Australia; ^2^ School of Psychological Sciences Macquarie University Sydney New South Wales Australia; ^3^ Department of Aged Care and Rehabilitation Liverpool Hospital Sydney New South Wales Australia

**Keywords:** attention, autobiographical memory, implicit memory, perception, spatial cognition

## Abstract

In 1941, André Rey published the Rey Complex Figure, a widely used test for assessing visual‐constructional ability and visual memory. It consists of two parts: copy and recall. Evaluating the copy portion presents challenges, as it requires the administrator to focus on both the process and outcome. The assessor must systematically track how the patient copies the figure in real‐time to evaluate their planning, organisation and executive abilities. This ‘clinician's copy’ serves as a record of the patient's approach, aiding later judgements about their cognitive skills. To ensure accuracy, clinicians need a method to record this process for later review or colleague consultation. This paper revisits Rey's suggestion of using different coloured pencils to observe the copy sequence, proposing an alternative. Instead of providing coloured pencils to the patient, we recommend that the administrator use them to record the copy sequence. This method aligns with test norms, reducing potential distractions for the patient while enabling both experienced and novice administrators to easily track and document the sequence of copying.

The Rey‐Osterrieth Complex Figure Test (ROCF) was originally developed by André Rey and extended by Paul‐Alexandre Osterrieth as a test capable of assessing visuo‐constructional ability and visual memory. In the 80 years since the two papers were originally published in French (Osterrieth, 1944; Rey, 1941) and subsequently translated to English (Corwin & Bylsma, 1993), the test has gone on to become one of the top 10 most administered assessments and, specifically, the most frequently administered test of visuospatial and visual‐construction ability by clinical neuropsychologists in the United States and Canada (Rabin et al., 2016). Rey described administration of the ROCF as consisting of two parts: copy and recall. In the copy phase, patients are presented with a complex, two‐dimensional geometrical figure that they are required to accurately copy. Time taken to copy the figure is recorded, although the task itself does not have a time limit. After a 3‐min delay, patients are asked to draw the figure from memory. The copy and recall phases assess visual‐constructional and visual memory abilities respectively. Subsequent administration protocols include a 30‐min delayed recall phase (Lezak et al., 2012; Strauss et al., 2006), allowing the clinician to comment on immediate versus delayed visual memory, as well as recognition trials that provide insights into visual memory encoding versus retrieval differences. Drawing accuracy of the elements and their locations are scored for both the copy and recall phases to generate quantitative measures of visual‐construction and visual memory.

Of focus in the current commentary is the copy stage of the ROCF. Rey described copying the complex figure as involving problem‐solving processes that require the patient to visually analyse the image in front of them, make sense of individual elements that join together to form larger coherent composites, and then draw these elements and composites to form the final figure. These abilities can be broadly classified under the umbrella term of visual‐construction. Crucially, visual‐construction ability not only involves attention, visual perception, fine motor skills and visuospatial analysis but also aspects of executive functioning (Shin et al., 2006). Put another way, the copy stage of the ROCF provides the clinician with an opportunity to evaluate the patient's ability to plan and organise their drawing as well as to detect possible perseverative responses.

The importance of reviewing patients' planning and organisational strategies underscores the process‐focused nature of the copy stage and the challenges that this brings. This is explicitly stated by Rey: ‘… the processes involved in making the copy… can be seen in the method the subject uses in making the copy. The problem for the examiner is to find a way to record the process objectively.’ (Corwin & Bylsma, 1993, pp. 4). Rey proposed an innovative solution to the issue of recording the drawing process, which involved giving patients seven or eight coloured pencils one at a time at various intervals to be used to complete their copy. Examiners could then follow the sequence of colours and review the qualitative visual‐construction planning and organisation post hoc.

Despite the simplicity and ingenuity of this approach, numerous clinicians continue to refrain from adopting Rey's method based on several limitations, highlighted in the Rey Complex Figure Test and Recognition Trial Professional Manual (Meyers & Meyers, 1995). The act of getting patients to switch pencils or markers can be overly distracting, especially for individuals with moderate‐to‐severe neurological dysfunction. Repeated switching may increase distractibility, utilisation behaviours and cognitive set maintenance difficulties (Stern & Prohaska, 1996), which may not only impact on patients' visual‐construction performance but also on memory encoding and subsequent retrieval in the recall phase. Patients with fine‐motor control and depth perception difficulties may also find it particularly challenging to switch between markers. Interestingly, Ruffolo et al. (2001) found evidence that patients who used the marker switching method produced faster and more accurate copies than those that generated a continuous copy with a single marker. The authors, however, caveat the results given that the dataset did not contain a statistically significant sample of patients with frontal lobe dysfunction who might be more susceptible to attention and inhibition difficulties. Furthermore, there are challenges with validating *when* markers should be switched. Rey recommended that colours should be changed at the discretion of the examiner, when they notice a shift in the organisational approach of the patient. In his view, clinicians with a clear understanding of the various ways in which patients approach the copy task would have little difficulty deciding when to switch colours. However, this approach places significant emphasis on clinical experience. Other authors suggest changes at fixed intervals (e.g. every 30 s; Bernstein & Waber, 1996). This approach, though reproducible, does not capture critical information pertaining to changes in organisational strategies occurring within the 30‐s blocks (Stern et al., 1999). Finally, the current test norms have not been developed using this multi‐colour approach. As stated by Meyers and Meyers (1995, pp. 7), ‘… the procedure of alternating colored markers is not used in the RCFT. Rather a #2 black lead pencil with a sharp point is used. Clinicians who wish to record the organisational and sequential aspects of the respondent's reproductions may follow along with the respondent and simultaneously draw the figure on the separate sheet of paper provided in the test booklet.’ This is noteworthy for a couple of reasons. One, the effects of any potential distractions caused by switching pens have not been factored into the quantitative score norms generated for copying or subsequent recall. Two, norms have also been developed for the time taken to complete the copy, which does not account for the additional time taken to switch markers. While the plausible impacts of shifts in attention on memory retrieval are clinically obvious, the relevance of the differences in time taken to complete the copy may not be immediately apparent. However, as highlighted by Meyers and Lange (1994), completion time can also be used to discriminate between neurologically impaired patients and healthy subjects.

Other methods of recording the copy process have since been developed. One approach is to take elaborate notes as suggested by Rey (Corwin & Bylsma, 1993), which Lezak et al. (2012) describes as simultaneously drawing a copy of the patient's work and sequentially numbering the individual elements. Another is to utilise a registration sheet containing a printed ROCF or a flowchart with multiple miniature copies of the ROCF (Lezak et al., 2012; Meyers & Meyers, 1995); the elements on the registration sheet are numbered and/or are annotated with arrowheads while, with the flowchart, individual elements are sequentially highlighted on each of the miniature ROCFs. These approaches also have their drawbacks. Numbering and drawing are challenging to do when patients are particularly speedy or when clinicians are early on in their training. The absence of different colours makes the final figure harder to recall the patient's approach to the task for further analysis when examining for planning and organisation and executive abilities. It is not the scope of this commentary to examine methods of analysing planning and organisation or the relative merits of these methods. It also is not the scope of this paper to examine the neurological or neuropsychological underpinnings of planning and organisation, and executive abilities. Simply what is being commented on here is that hitherto available methods for recording client or patient approaches to the copying of the figure can be cumbersome and overly distracting for the clinician and the patient. The clinician needs a simple method of recording how the patient approached and completed the task which can be used as a reminder when examining the patient's original copy for further analysis of planning and organisation and other errors or omissions and what these may mean neuropsychologically and diagnostically. The use of a flowchart does not generate one complete picture. Qualitative observations of the organisation of the whole figure and/or its composite is increasingly challenging; both the flowchart and registration sheet are unable to account for any additions or oddities in the drawings patients produce. No one method will be able to record all aspects of the patients approach to the task with omissions or perseverations or additions. However, a more straightforward method is needed that is unobtrusive for patients and simple for the clinician. Importantly the clinician must maintain their focus on the patient's performance, watching all aspects of their approach to the task. As such any approach for recording the copy by the clinician must be unobtrusive and not distracting for the clinician. More recently, technological advances such as the use of tablets to record individuals drawing strokes serve to overcome the limitations outlined above but lack the accessibility and affordability that comes with paper‐and‐pencil administration (Kim et al., 2020). Technology is not always available and is expensive. Again, a simple and elegant nonintrusive method that is not distracting for either the clinician or the patient must be found.

Of the various methods available to record the copy process, Rey's suggested use of coloured markers has clear advantages in terms of allowing the clinician to easily record and visualise a patient's visual‐construction and approach to the task. It is a reminder to the clinician and a documented record of the patient's approach and can then be used to compare against the patient's original copy for further analysis of planning and organisation and other important aspects of neuropsychological analysis, such as executive functioning. As previously alluded to, it is not the scope of this commentary to provide an in‐depth analysis of the practicalities and theoretical or neurological underpinnings of planning and organisation or executive functioning; however, detailed conceptual frameworks for such analysis and underpinnings can be found elsewhere (Adams & Gabel, 2014; Libon et al., 2022; Poreh, 2000). Using coloured pens has significant benefits when it comes time to reviewing the copy and commenting on the patient's qualitative approach to visual construction and/or using available qualitative scoring systems (e.g. The Boston Qualitative Scoring System; Boone, 2000; Somerville et al., 2000; Stern et al., 1999). However, the confounding variables generated by increasing patients' cognitive load on executive functioning are likely to continue to deter clinicians from embracing this method. One way to minimise the limitations posed by this approach is for clinicians, rather that patients, to make use of the coloured markers to track visual‐construction planning and organisation. In this scenario, the patient is given a single marker (or a #2 black lead pencil)—from start to finish—and is instructed to copy the complex figure. As the patient copies the figure, the clinician simultaneously renders the patient's copy, switching coloured markers at times when the patient makes noteworthy changes in the organisation of their drawing. Changes in coloured pens by the clinician can be discretionary to the clinician or according to some systematic qualitative method. However, it is the clinician that changes the coloured pens. The advantages of this approach are apparent: the clinician renders a real‐time version of the patient's copy that is easy to record and documents the patient's approach to the task without adding to the administration time or cognitive/attentional load placed on the patient. The clinician then has a self‐rendered copy that is an immediate visual reminder of the patient's approach. This method reduces the load on the clinician relative to other methods that have been documented as potential ways of rendering patient copies. The method discussed here allows the clinician to maintain their focus on the patient and their approach and performance on the task. Importantly, the clinician also has the opportunity, either at the same time or after the patient has completed a task, to record any errors (in different coloured pens if they wish) or omissions or behavioural observations pertinent to future analysis of the patient's copy. Further, it is easy for the clinician to present both their copy and the patient's original one to peers when receiving consultation/supervision or making a case presentation. Crucially, the copy completed by the patient remains consistent with the standard administration protocol in the professional manual, which allows the clinician to then utilise quantitative normative data with greater certainty. That is, patients complete their copy in line with standardised administration procedures. The recording method by the clinician remains unencumbered and is much simpler than currently documented by other methods. It means that all normative data applicable to the patient's copy can be utilised with confidence maintaining validity and reliability.

This approach is not without its limitations. It still does not resolve the issue of *when* the clinician should switch markers but does leave it open to clinical discretion and discernment based on how and when the clinician would like to note significant changes or aspects of the patients approach, and is reliant on their experience. Clinicians can also choose to switch pens according to qualitative behavioural scoring methods (e.g. The Boston Qualitative Scoring System). However, even the novice clinician can be confident that the timing of the decision is likely to have no impact on the copy performance of the patient. Anecdotally, this has been our experience in an inpatient aged care setting assessing patients with significant and severe cognitive difficulties, including in the context of evaluating decision‐making capacity. Another potential pitfall involves the use of coloured pens themselves, both for clinician and patients. It has been our experience, and it is anticipated that, for the clinician there should be minimal initial increase in the cognitive load from tracking and rendering the patient's drawing, and seamlessly reaching for and switching pens. Preparation will be key to have, for instance, either a set of coloured pencils or uncapped pens, placed in their lap or within easy reach. Clinicians may consider using a single multicoloured ballpoint pen for ease of administration. These pens have 4–6 colours, which should be sufficient for a single administration. For reliable use across clients, the clinician can always choose to start with the same colour (e.g. red) and then click‐and‐change colours in a clockwise direction to easily keep track of the order of colours chosen. If using individual colour pens/pencils, clinicians can make a tally mark with each colour, on the top corner of the sheet, to keep track of the colour order. For the patient, it is unlikely if they will notice the clinician switching/clicking of different coloured pens, and/or how that might impact on their performance, in terms of serving as a distraction. Our own utilisation of this method in clinical practice—including in a geriatric population displaying a variety of dysexecutive features and severe cognitive impairments—has yet to yield a scenario in which the patient shows signs of noticing or becoming distracted. We, therefore, predict that this method can be reliably used while limiting potential confounds to patient testing performance, as well as being much more efficient and effective for the clinician. As with all testing administration, however, the clinician must do their best to be discrete in how they record the patient's performance.

The method suggested here sits within the process approach framework and acknowledges the benefits and importance of the clinician's capacity to reliably and validly provide analysis and comment on qualitative aspects of the patient's performance on this task. As most clinicians are aware, the planning and organisational analysis and other executive aspects of a patient's approach to the Rey Complex Figure has become clinically very significant for differential diagnosis, rehabilitation purposes, and depth of understanding of a patient's executive presentation (Ogino et al., 2009; Shin et al., 2006; Watanabe et al., 2005; Zhang et al., 2021). Whilst perhaps not an original idea, it is certainly in one of the author's (AB) extensive experience of many decades of practice that this simple technique has never been discussed before nor is it documented. The clinical implications of applying a process approach method of analysis to this task cannot be overstated and is well reported in the literature (Zhang et al., 2021). Some notable examples of the impact of such an approach on differential diagnosis and clinical practice include differentiating between early‐ and late‐onset Alzheimer's disease (Kim et al., 2020), Parkinson's Disease and Progressive Supranuclear Palsy (Tommasini et al., 2023), as well as the different information processing styles of older autistic adults (Davids et al., 2020).

One practical follow‐up from this commentary will be to empirically evaluate the clinical utility of this proposed method and compare it to some of the existing methods outlined earlier. Clinicians should be recruited to provide qualitative and quantitative feedback regarding ease of use and administration across these methods and the clinical implications and impact on diagnostic outcomes.

Through this commentary, we hope that more clinicians are encouraged to use Rey's copy recording method put forward in his 1941 paper. The subtle tweak to administration proposed here acknowledges the merits of the method developed by Rey (1941) while reframing it to improve the quality and ease of clinical administration, as well as its alignment with existing normative data. An awareness of the history of cognitive testing and its application in current contexts is a unique requisite of knowledge‐based assessment competencies for neuropsychologists (Wong et al., 2024). This is true for clinicians across educational levels and career stages. Arming clinicians with simple tools that can be effectively used to assess health conditions can also go far in supporting practitioners in resource‐poor countries where brain‐related conditions are expected to increase and psychological practice continues to have minimal regulation (Bedi et al., 2021; Owolabi et al., 2023).

## AUTHOR CONTRIBUTIONS

**Maneesh V. Kuruvilla:** Conceptualization; writing – original draft; methodology. **Angela Blazely:** Writing – review and editing; supervision.

## CONFLICT OF INTEREST STATEMENT

The authors declare no conflicts of interest.

## Data Availability

Not applicable.
